# Primary dermoid cysts of the cecum: A systematic review and case report

**DOI:** 10.1097/MD.0000000000048521

**Published:** 2026-05-08

**Authors:** Amagd Elsmani, Ali Toffaha, Hamza A. Abdul-Hafez, Wael Al-Ahmad, Mahmood Al-Dhaheri, Mahwish Khawar, Mohammed Yousif, Mohammed Kurer

**Affiliations:** a Department of General Surgery, Hamad General Hospital, Doha, Qatar; b Colorectal Surgery Unit, Department of Surgery, Hamad General Hospital, Doha, Qatar; c Department of Medicine, College of Medicine, QU Health, Qatar University Doha, Qatar; d Department of Medicine, Faculty of Medicine and Health Sciences, An-Najah National University, Palestine.

**Keywords:** case report, Cecum, Colorectal, Dermoid, teratoma

## Abstract

**Rationale:**

Cecal dermoid cysts, mature cystic teratomas, are exceedingly rare. They may present with nonspecific abdominal pain and mimic common surgical conditions. We report a pediatric case of cecal dermoid cyst presenting with acute appendicitis and provide a systematic review of all published cecal dermoid cases.

**Patient concerns:**

A 14-year-old female presented with 1 day of right lower abdominal pain, nausea, vomiting and anorexia; she had prior vague right-sided pain with an ultrasound 5 years earlier that identified a right iliac fossa cyst, which was not followed up.

**Diagnoses:**

Contrast-enhanced computed tomography showed an inflamed appendix with peri-appendiceal fat stranding and an appendicolith, and a separate well-defined hypodense lesion (3.5 × 3.2 cm) at the ileocecal junction. Differential diagnoses included duplication cyst, dermoid cyst, or low-grade mucinous neoplasm.

**Interventions:**

Diagnostic laparoscopy confirmed acute appendicitis and a distinct cecal mass with enlarged ileocolic lymph nodes. Because malignancy could not be excluded intraoperatively, a laparoscopic oncologic right hemicolectomy with ileocolic anastomosis was performed.

**Outcomes:**

Histopathology demonstrated a benign dermoid cyst and acute suppurative appendicitis with microscopic perforation; 20 reactive lymph nodes were negative. The postoperative course was uneventful and the patient was discharged.

**Lessons:**

Cecal dermoid cysts are rare and can mimic common pathologies such as appendicitis or ovarian masses. Preoperative diagnosis is challenging; definitive diagnosis requires surgical excision and histopathology. Laparoscopy is a useful diagnostic and therapeutic tool, but oncologic resection may be required when malignancy cannot be excluded intraoperatively.

## 1. Introduction

Dermoid cysts, also known as mature cystic teratomas, are exceptionally rare extragonadal lesions; they are benign tumors with elements derived from all 3 germ layers. They can be classified as congenital or acquired.^[1-3]^ Acquired intra-abdominal dermoid cysts may result from previous surgery or trauma, potentially due to implantation of cutaneous tissue into the peritoneal cavity.^[4]^ Conversely, congenital dermoid cysts are believed to arise from ectodermal implantation during embryogenesis, particularly at the time of neural groove closure during development.^[5]^

While dermoid cysts are more common in the gonads and midline structures, such as the mediastinum, anterior neck, and central nervous system, abdominal and namely cecal dermoid cysts remain extremely rare.^[6,7]^ To our knowledge, only seventeen cases have been reported, and our case was the only one presenting with concurrent appendicitis. The clinical presentation of cecal dermoid cysts is often mistaken for more common conditions, hence reporting of such a rare condition and its presentations is essential for understanding its origin, pathological behavior, and optimal management strategies.^[3,5,7]^

We report a case of pediatric cecal dermoid cyst presenting concurrently with acute appendicitis, an uncommon presentation that can obscure preoperative recognition. In addition to report this rare entity, we conducted a comprehensive review of the cecal dermoid cyst including all reported cases in the literature to provide the most comprehensive contemporary summary of demographics, imaging features, operative strategies and outcomes.

## 2. Methods

This systematic review performed in accordance with the Preferred Reporting Items for Systematic Reviews and Meta-analysis guidelines,^[8]^ focusing on collecting and summarizing all published case reports and case series of cecal dermoid cysts.

### 2.1. Searching process

**Figure 1.**
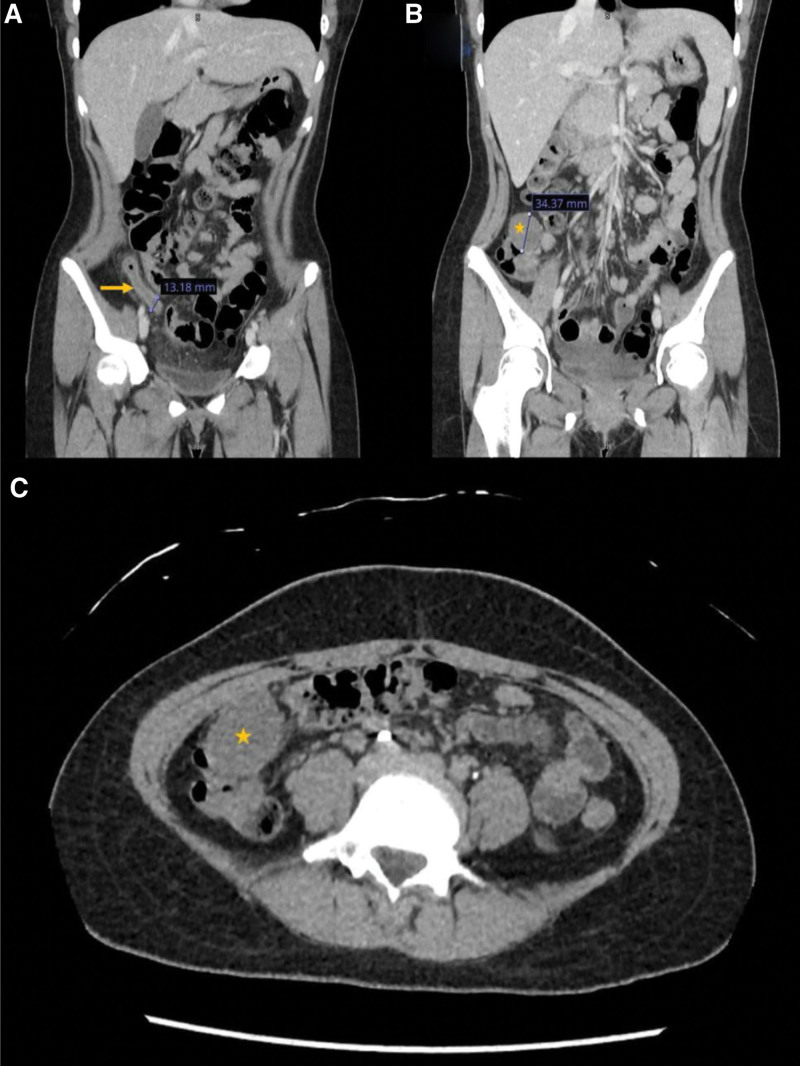
CT scan of the abdomen (A) Coronal view showing inflamed appendix (arrow) with enlarged thick appendix and peri-appendiceal fat stranding. (B, C) are coronal and cross sectional views respectively showing the cecal mass (star).

We conducted a comprehensive search from inception until October 2025 in PubMed to find all eligible published cases that could be included in our study. We searched using the Medical Subject Headings (MeSH) keywords: ((“cecum” OR “cecal” OR “caecal”) AND (“dermoid cyst” OR “mature cystic teratoma” OR “cystic teratoma” OR “dermoid” OR “teratoma”)). The search strategy and study selection process were illustrated using the Preferred Reporting Items for Systematic Reviews and Meta-analysis 2020 flowchart (Fig. 1).

### 2.2. Studies eligibility and inclusion

We included case reports and case series on patients with cecal dermoid cysts of any age or gender, with no restriction on publication date. Only English-language reports were considered. Other study designs, animal research, and articles published in other languages were excluded. We also excluded reports where the dermoid cyst was located in other than the cecum, or where critical clinical or surgical data were missing.

All titles and abstracts were screened independently by 2 authors. Full texts were reviewed for probably eligible articles, and any disagreements were resolved by senior author.

### 2.3. Data extraction

Two independent authors extracted data from the final included studies using a standardized Microsoft Excel sheet. Any conflicts were resolved by senior author. We collected the following variables for each patient: study characteristics (country, year) and patient demographics including sex, age, and past medical history. Presentation data included symptoms, physical examination findings, and misdiagnosis. Tumor characteristics included size, attachment, gross appearance, and contents, along with histopathological details such as diagnosis and histological subtype. Imaging modalities assessed, including computed tomography (CT) findings, as well as colonoscopy, ultrasound, and magnetic resonance imaging features. Management details covered surgical intervention type, postoperative complications, and outcomes (mortality, recurrence, cure).

### 2.4. Data analysis

Data were analyzed descriptively. Categorical variables such as gender, symptoms, and surgical details were summarized using frequencies and percentages. Continuous variables like age and tumor size were reported using mean, median, and range, where appropriate.

## 3. Case presentation

A 14-year-old female with no prior medical or surgical history presented to the emergency department with a 1-day history of abdominal pain, associated with nausea, vomiting, and anorexia. She reported vague episodes of right-sided abdominal pain, for which she had an abdominal ultrasound 5 years ago which showed a right iliac fossa cyst, she was referred for further investigations but she traveled at the time and missed her follow up. She denied changes in bowel habits, urinary symptoms, weight loss, or bleeding per rectum, and there was no family history of malignancy.

On examination, she was febrile (38°C) and tachycardic (heart rate 100 bpm) but maintained stable blood pressure (115/70 mm Hg) and oxygen saturation on room air. She appeared thin and ill-looking. Abdominal examination revealed right iliac fossa tenderness with rebound tenderness, without palpable mass, guarding, or rigidity. Laboratory investigations demonstrated leukocytosis (WBC 12 × 10^3^/μL), anemia (hemoglobin 8 g/dL), and a C-reactive protein level of 10 mg/L.

A contrast-enhanced CT scan of the abdomen revealed a dilated, fluid-filled appendix measuring approximately 9 mm in maximum caliber with enhancing wall, peri-appendiceal fat stranding and an appendicolith near its base. Additionally, a well-defined hypodense lesion measuring 3.5 × 3.2 cm was identified near the ileocecal junction, close to the base of the appendix. Multiple small lymph nodes were noted along the root of the mesentery and in the right iliac fossa, the largest measuring 7.3 mm. The radiological differential diagnosis included acute appendicitis, additionally, a cecal lesion that could be duplication cyst, dermoid or low-grade mucinous neoplasm (Fig. 1).

**Figure 2.**
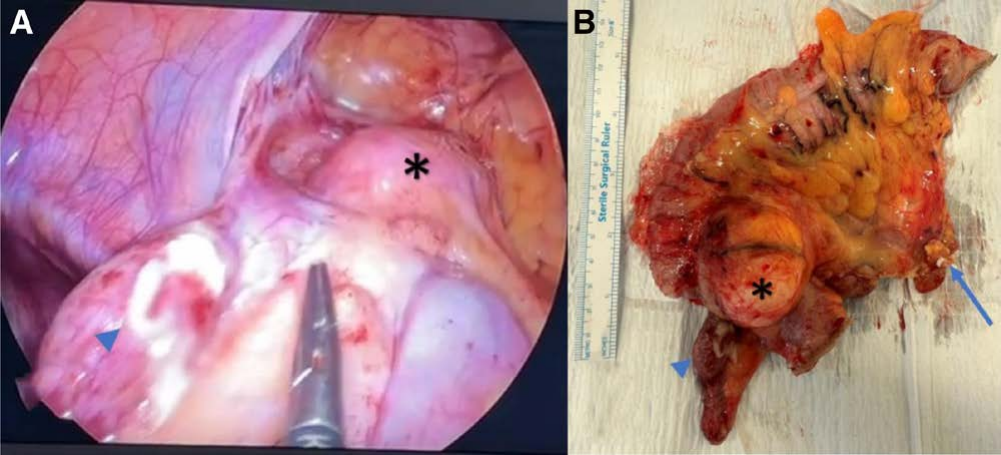
(A) Intra-op laparoscopic image and (B) post op specimen picture showing acute appendicitis (arrow head) with separate cecal lesion (asterisk), the arrow shows the central vascular ligation of ileocolic pedicle.

Following initial resuscitation, the patient underwent diagnostic laparoscopy. Intraoperatively, the appendix was inflamed with a healthy base, and a separate cecal mass was observed at the ileocecal junction, along with enlarged lymph nodes along the ileocolic pedicle. There were no fat creeping or other visible lesions (Fig. 2). A laparoscopic oncological right hemicolectomy with ileocolic anastomosis was performed.

Histopathological examination revealed a benign dermoid cyst, completely excised, and acute suppurative appendicitis with transmural inflammation and necrosis, with periappendicitis suggestive of microscopic perforation. No viable mucosa was identified in the appendix. Twenty benign reactive lymph nodes were examined (0/20 positive for malignancy), and the proximal and distal resection margins were viable and free of dysplasia or malignancy.

The postoperative course was uneventful, and the patient was discharged on postoperative day 4 without complications. She was followed up at 2 weeks, 3 months, and 1 year postoperatively, and remained well and asymptomatic, with no evidence of recurrence or other complications.

## 4. Results

### 4.1. Patients selection

**Figure 3.**
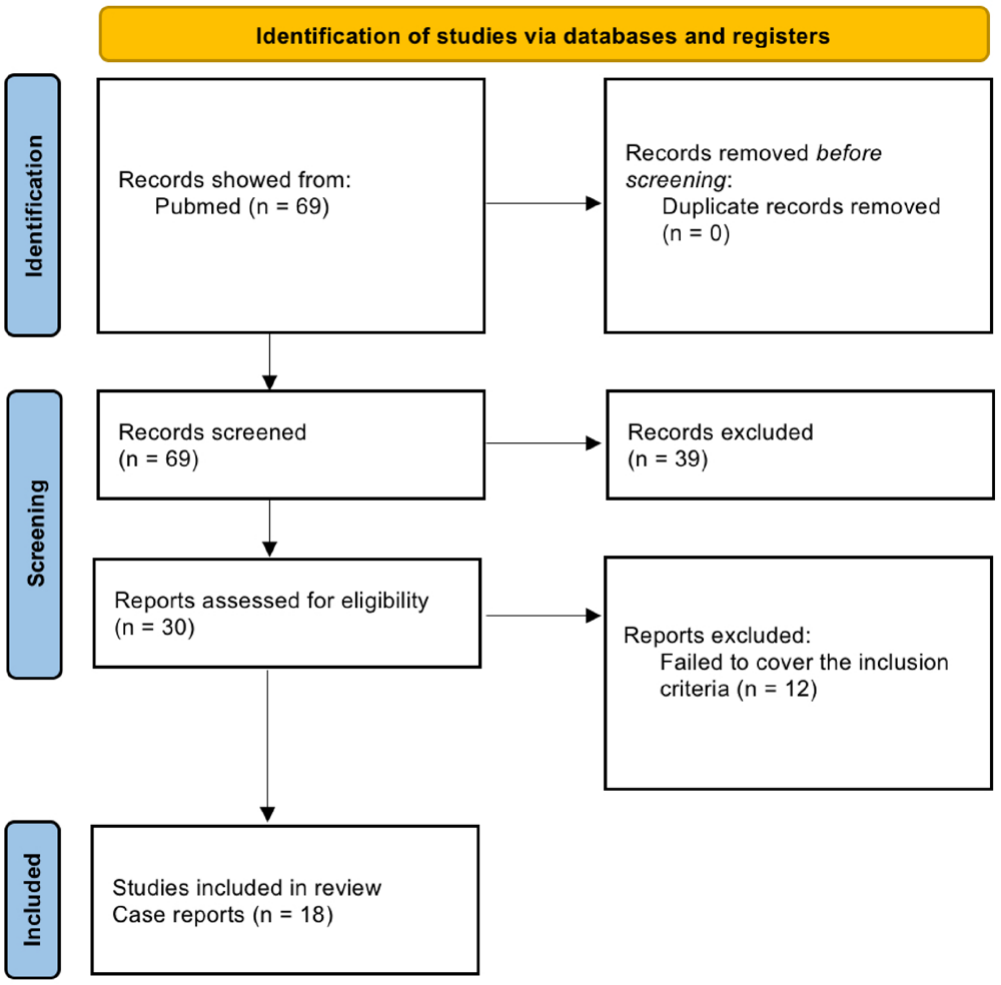
PRISMA flowchart.

Our search process from Pubmed retrieved 69 records. Thirty nine articles were excluded after title and abstract screening due to multiple reasons, including language, irrelevance, or incorrect study design, resulting in 30 full-text articles for detailed review and eligibility assessment. After full-text screening and evaluation, 12 failed to cover the inclusion criteria and thus were excluded. Therefore, 18 articles were included as final studies for data extraction^[1,3,5,7,9-21]^ (Table 1, Fig. 3).

**Table 1:** Summary of the reported cases of cecal dermoid cysts.

	Study characteristics		Patient demographic		Presentation		Tumor				Histopathology	Imaging	Management	
	Study ID*.Year	Country	Sex (F/M)	Age (years)	Presenting symptoms	Physical exam	Size (cm)	Attachment	Gross appearance	Content	Histopathological details	CT/U.S	Surgery	Outcome (mortality, recurrence, cure)
1	Current study (2025)	Qatar	F	14	Lower abdominal pain, vomiting	RLQ tenderness	3.5 × 3.2	Iliocecal junction	NR	NR	NR	CT: well-defined Hypodense lesion	Laparoscopy + Right hemicolectomy	Cure
2	Haddadi et al (2024)	Algeria	M	36	Asymptomatic	Tenderness in right iliac fossa	10 × 8	Opposite the cecum	Soft, smooth, thin wall	pasty material	Atrophic sq.	CT: intraperitoneal cystic mass, measuring 71 × 56 mm in long axis, extending over 93 mm. located opposite to the cecum and cleaved from it. No partitions or calcifications. Ileoscopy UR	Laparotomy + Cystoctomy	Cure
3	Bilen et al (2024)	Turkey	F	35	Lower abdominal pain, constipation	RLQ tenderness	10 × 7 × 7	Base of cecum	Solid, yellow, thick wall	Solid and cystic components, keratinous substance	Sq. non malignant, giant cell reaction	U.S: transvaginal ultrasonography: semisolid heterogenous mass lesion with a size of 105 × 77 mm originating from the right lateral uterus	Laparotomy + Ileocecal resection	Cure
4	Gellings et al (2023)	United States	M	66	Lower abdominal pain	NR	4.5 × 3.5 × 2.6	Lateral wall of cecum	Soft, no solid areas, no muscular connection, encased in fat	NR	NR	CT: apparent extraluminal mass abutting the cecum and appendix	Laparoscopy + Partial cecetomy + Appendectomy	Cure
5	Mishra TS et al (2021)	India	F	35	Lower abdominal pain	Well-defined mass in RLQ	10 × 7 × 5	Medial wall of cecum	NR	NR	Strtf. Sq., pilosebaceous units	CT: well-defined hypodense lesion in the right lower abdomen U.S: 10 × 5 sized, well-defined solid cystic lesion with calcification in the right iliac fossa	Laparoscopy + cystectomy	Cure
6	Kamdem et al (2019)	Switzerland	M	44	Asymptomatic	UR	6.1 × 4.9 × 4.9	Bottom of ileocecoal valve	Cylindrical in shape with smooth outer surface	Tan-brown cheese-like material	Kering. Strtf. Sq., focal sebaceous, sm. muscles continuous with musc. propria	CT: well-defined cystic mass with No greasy substance or calcification, thin wall, in contact with the appendix and developing on the bottom of the ileocecal valve. appendix is normal no fluid collection ot lymphadenopathy	Laparoscopy + Right hemicolectomy	Cure
7	Destro et al (2019)	Italy	M	2	Distended abdomen	Abdominal distention	NR	Left colon wall	NR	NR	Sq. and glands without atypia	CT: situs ambiguous with left liver and asplenia, horseshoe kidney and oval shaped mass	Laparotomy + cystectomy	Cure
8	Nahidi A et al (2016)	Iran	F	41	Lower Abdominal pain	Moveable non-tender mass in RLQ	10 × 10	Cecal wall muscularis propria	NR	Keratin	NR	CT: a 10 × 10 cm pelvic mass in the vicinity of the right ovary. U.S: a 10 × 10 cm pelvic mass in the vicinity of the right ovary	Laparotomy + Cecetomy + Mikulicz colostomy	Cure
9	Lao et al (2014)	United States	F	16	Asymptomatic	NR	5 × 3.7 × 3.2	Base of cecum	Soft, Smooth mass	Hair shafts, keratinous debris	Kering. Sq., sebaceous glands, hair follicles, eccrine ducts	CT: 5 cm pelvic Cystic mass	Laparoscopy + Cystectomy	Cure
10	Kirli et al (2013)	Turkey	M	11	Lower Abdominal pain	UR	3 × 3 × 2	Mesentry of ileocecoal region	Soft, lipomatous in appearance, thin wall	Pilosebaceous units, mature adipose tissue, Keratin	NR	CT: well circumscribed cystic mass between intestinal loops, thin wall and consistent material. U.S: well circumscribed cystic mass between intestinal loops, thin wall and consistent material	Laparoscopy + cystectomy with adjacent lymph node	Cure
11	Schuetz and Elsheikh (2002)	United States	M	30	Right upper Abdominal pain	Palpable non-tender mass in right upper abdomen	8 × 6 × 5	Mesentric border of cecum	Firm, thin wall, smooth lining	Tan to white cheesy material	Anucleated and benign nucleated Kering. Stratf. Sq.	CT: nonenhancing, well-marginated mass seperate from the liver with an impreceptible wall. U.S: prominent mass inferior to the right lobe of the liver, 7 × 6.34, heterogenous echogenic pattern and no internal echoes	Laparotomy + ileocolic resection + appendectomy	Cure
12	Nirenberg et al (2001)	Australia	M	34	Lower Abdominal pain	Tender RLQ mass	7.5 × 6 × 5	Lower border of the cecum	Firm, mostly covered by serosal surface	Soft cream to pale tan coloured material	Kering. strtf. sq. with a granular layer, occasional skin appendages, giant cell, keratinous material, smooth muscle (deep to the surface)	U.S: well-defined mass approximately 5 cm in size in the right iliac fossa of uncertain origin	Laparotomy + cystectomy with appendectomy	Cure
13	Fujita et al (2001)	Japan	F	26	Asymptomatic	NR	5.4 × 4.8 × 3.5	Subserosal tissue of cecum and involved in ileocecal region	Well curcunscribed mass, thin wall	White oily material	Ker. strtf. sq., hair follicles, sebaceous glands	NR	Laparotomy + Ileocecal resection	Cure
14	Mellado et al (2000)	Spain	F	39	Lower abdominal pain and destension	NR	20 × 16 × 6	Medial side of cecum, appendix	Brilliant, smooth	Creamy white material	Epidermoid epithelium with occasional sebaceous glands	CT: well-defined homogenous mass. A thin capsule noted. cyst was seen to abut the cecum, uterus, left broad ligament, and the iliac vessels. Slight displacement of abdominal wall. U.S: well-defined moderately inhomogenous slightly hyperechoic lesion. Located in the pelvic region anterior to the uterus	Laparatomy + Partial cecectomy + appendectomy	Cure
15	Wikinson et al (1995)	UK	F	34	Lower abdominal pain, vomiting, and constipation	Firm tender partially cystic abdominopelvic mass	10 × 6 × 6	Serosa of cecum, adjacent to ileocecal valve	Smooth, thin wall	Pultaceous material	Kering strtf. sq.	U.S: heterogenous mass in the right adnexal structures with a cystic oomponent	Laparatomy + Right hemicolectomy	NR
16	Mossey et al (1977)	United States	F	53	GI bleeding	NR	4	NR	Irregular, polypoid	Blood coagulum	Strtf. sq. and chronic inflm. cells	NR	Laparotomy + Right hemicolectomy + Ileotransverse colostomy	Cure
17	Finlay-Jones et al (1973)	Australia	M	28	Asymptomatic	Mobile non-tender mass in right iliac fossa	5	Anterior wall of cecum	Smooth, uniform	Pale sebacous material, white hairs	Strtf. sq. with well developed stratum granulosum with prominent keratohyaline granules. lost ep. replaced by gran. tiss. and immune cells.	NR	Laparotomy + Right hemicolectomy + appendectomy	NR
18	Kay (1971)	United States	F	1	Asymptomatic	Non-tender right side mass	8	Medial side of cecum	Grey internal surface, covered with white flecks of keratin-like material	NR	Strtf. Sq., Hair follicles and sebacous glands in the wall, giant cell reaction.	X-Ray (upper gastrointestinal series, intravenous pyelogram, and barium enema): revealed a smoot mass, not attached to the kidney, in the upper and lower mesentry	Laparotomy + ileocolic resection + Ileal ascending colostomy	Cure

* For space considerations, only the first author is cited.

D = duration of symptoms, d = days, h: hour/s; Hb = hemoglobin in mg/dL; Histo = histology, Kering = Keratinizing, L = left, M = male, m = month/s, NK = not known, NR = not reported, PH = past histories, PE = physical examination, R = right, UR = unremarkable, w = week/s; y = year, Sq = squamous epithelium, Strtf = Stratified.

### 4.2. Patient’s demographic and tumor characteristics

Eighteen patients were included in our review. The mean age was 30.1 ± 18.8 years (median 32 years, range 1–71). Ten patients (55.6%) were female and 8 (44.4%) were male. The largest proportion originated from the United States (27.8%); 2 patients were from Turkey (11.1%) and 2 from Australia (11.1%); the remaining 9 patients (50.0%) came from other countries (1 case each from Qatar, Switzerland, India, Italy, Iran, the UK, Spain, Algeria and Japan). Tumor size had a mean of 7.66 ± 4.20 cm with a median of 7.1 cm (range 3–20 cm; Table 2).

**Table 2:** Patient demographics and tumor characteristics (n = 18).

Variables	Result (Total = 18)
Age (yr)	
Mean (± SD)	30.1 (18.8)
Median (range)	34 (1–71)
Gender	
Male, n (%)	8 (44.5)
Female, n (%)	10 (55.5)
Country	
United States, n (%)	5 (27.8)
Turkey, n (%)	2 (11.1)
Australia, n (%)	2 (11.1)
Others (Qatar, Switzerland, India, Italy, Iran, UK, Spain, Algeria, Japan), 1 for each, n (%)	9 (50)
Size (cm)	
Mean (± SD)	7.66 (4.2)
Median (range) largest	7.5 (3–20)

SD = standard deviation.

Attachment site was reported in 17 of 18 cases. The most common site was the ileocecal region, (including the iliocecal junction, ileocecal valve, adjacent serosa/subserosal tissue of the ileocecal region) in 5 patients. The medial wall of the cecum was the attachment site in 3 patients; one of these cases also involved the appendix. The base of the cecum was reported in 2 patients. Two tumors were attached to the contralateral/left colonic wall or described as “opposite the cecum.” Less commonly reported sites, 1 for each, were the lateral wall of the cecum, the lower border of the cecum, the anterior wall of the cecum, the mesenteric border, and muscularis propria location within the cecal wall (Table 1).

The content of cecal dermoid cysts was generally described as keratinous or sebaceous material, often noted as tan to white cheesy, creamy, pasty, or oily in appearance. In several cases, hair shafts, pilosebaceous structures, and mature adipose tissue were observed, reflecting the typical ectodermal and mesodermal components of mature cystic teratomas. Some lesions demonstrated both solid and cystic components with keratinous debris, while others contained soft cream-colored or pultaceous material. A few reports mentioned atypical findings such as blood coagulum.

### 4.3. Clinical features and diagnostic modalities

Clinical presentation was variable. The most common complaint was lower abdominal pain (n = 9, 50%); 6 patients (33.4%) were asymptomatic and diagnosed incidentally. Constipation and abdominal distension were each reported in 2 patients (11.1%); upper abdominal pain and gastrointestinal bleeding were each reported in 1 patient (5.6%). On examination a palpable mass was present in 10 patients (tender in 5 patients; non-tender in 5; Table 3).

**Table 3:** Clinical presentation, diagnostics, surgical management and outcomes (n = 18).

Variables	Result total = 18
Clinical presentation	
Lower abdominal pain, n (%)	9 (50)
Asymptomatic, n (%)	6 (33.4)
Constipation, n (%)	2 (11.1)
Distended abdomen, n (%)	2 (11.1)
Upper abdominal pain, n (%)	1 (5.5)
GI bleeding, n (%)	1 (5.5)
Examination findings	
Tender mass, n (%)	5 (27.8)
Non-tender mass, n (%)	5 (27.8)
Misdiagnosed as	
Ovarian mass, n (%)	2 (11.1)
Appendicular mucocele, n (%)	1 (5.5)
Meckel’s diverticulum, n (%)	1 (5.5)
Uterine myoma, n (%)	1 (5.5)
Diagnostic modality	
CT, n (%)	11 (61.1)
Histopathology, n (%)	14 (77.8)
Ultrasound, n (%)	8 (44.5)
MRI, n (%)	2 (11.1)
Surgical approach	
Laparoscopy, n (%)	6 (33.4)
Laparotomy, n (%)	12 (66.7)
Surgery	
Ileocecal/ileocolic resection, n (%)	4 (22.2)
Right Hemicolectomy, n (%)	5 (27.8)
Cystectomy, n (%)	6 (33.4)
Partial cecectomy/cecetomy, n (%)	3 (16.7)
Stoma	
Yes, n (%)	3 (16.7)
No, n (%)	15 (83.3)
Outcome	
Cure, n (%)	16 (88.9)
Dead, n (%)	0 (0)
NR, n (%)	2 (11.1)

CT = computed tomography, MRI = magnetic resonance imaging, NR = not reported.

Preoperative CT scan was the commonest imaging modality (n = 11, 61.1%); ultrasound was used in 8 patients (44.5%) and magnetic resonance imaging in 2 (11.1%). Histopathological diagnosis was confirmed in 14 patients (77.8%). Common preoperative misdiagnoses included ovarian mass (n = 2), appendicular mucocele (n = 1), Meckel’s diverticulum (n = 1) and uterine myoma (n = 1; Table 3).

### 4.4. Surgical management and outcome

**Figure 4.**
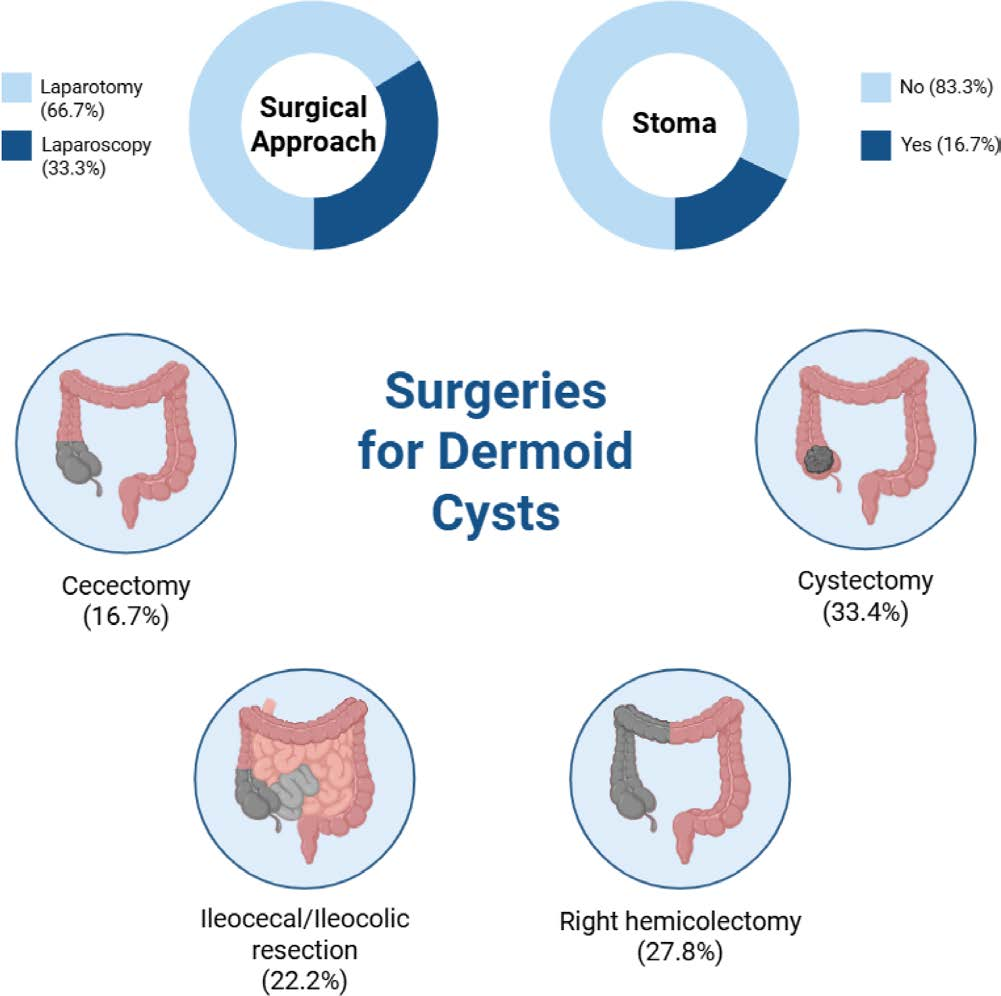
Surgical management of the cecal dermoid cysts. Created in BioRender.

The operative approach was predominantly open: laparotomy in 12 patients (66.7%) and laparoscopy in 6 (33.3%). Procedures performed included ileocecal/ileocolic resection (n = 4), right hemicolectomy (n = 5), cystectomy (n = 6) and partial cecectomy/cecetomy (n = 3). A stoma was used in 3 patients. Sixteen patients were reported as cured. There were no deaths in the included cases (Fig. 4, Table 3).

## 5. Discussion

Epidermoid and dermoid cysts of the cecum are remarkably rare, with only a few cases reported in the literature (Table 2).^[6,22]^ While dermoid cysts have been documented in various internal organs such as the testis, epididymis, spleen, liver, and appendix,^[2,4]^ their pathogenesis remains an area of ongoing investigation. One proposed mechanism is the formation of sequestration cysts, which may be either congenital or acquired.^[23,24]^ Other theories, including serosal metaplasia induced by chronic inflammation or heterotopic tissue inclusion during embryonic development, have also been suggested, but none fully explain the occurrence of these cysts within the cecum, particularly in patients with no history of surgery or trauma.^[1,6]^

**Figure 5.**
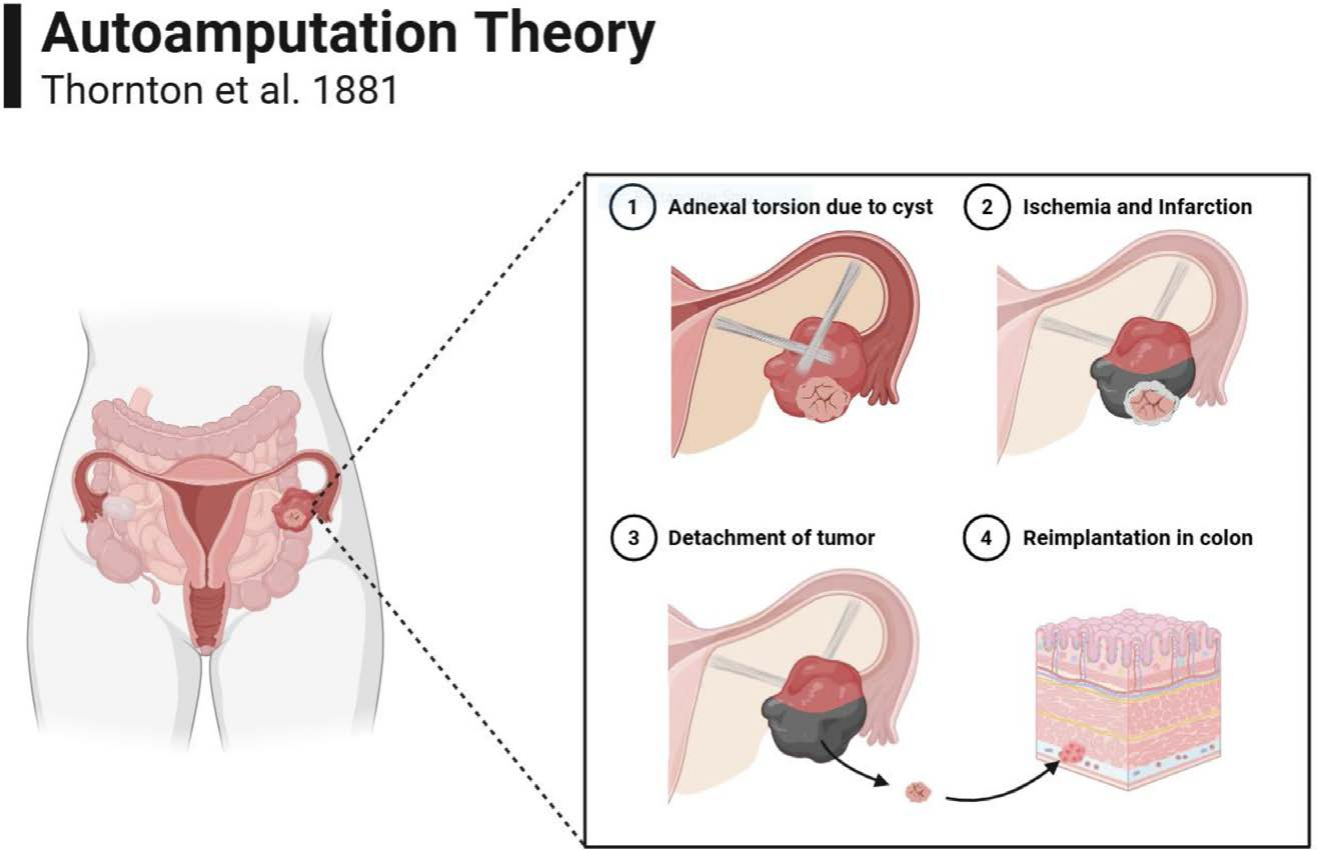
Thornton Autoamputation theory for dermoid cysts formation in the abdomen. (1) Due to cystic mass, adnexal torsion takes course, cutting blood supply and causing edema and enlargement of the affected ovary. (2) As blood supply is cut, the ovarian and cystic tissues infarct and undergo necrosis. (3) Necrosis destabilizes the connection between the cyst and the normal tissue; thus, a portion of the cyst break off and escape into the abdomen. (4) Cyst reimplants in the subserosal layer of the colon, where it continues growing. Created in BioRender.

Acquired dermoid cysts are generally believed to arise from the seeding of cutaneous tissue via trauma or intra-abdominal surgery, a mechanism widely accepted in the literature. However, the pathogenesis of congenital dermoid cysts, especially in the cecum, remains less understood. Among the theories proposed for the formation of abdominal dermoid cysts, the most widely accepted is Thornton’s Autoamputation Theory.^[1]^ Thornton postulated that extragonadal teratomas, often originating from ovarian tissue, become detached due to adnexal torsion, causing infarction and necrosis. The detached tissue is then reimplanted elsewhere in the abdominal cavity, such as the cecum (Fig. 5).^[25]^

**Figure 6.**
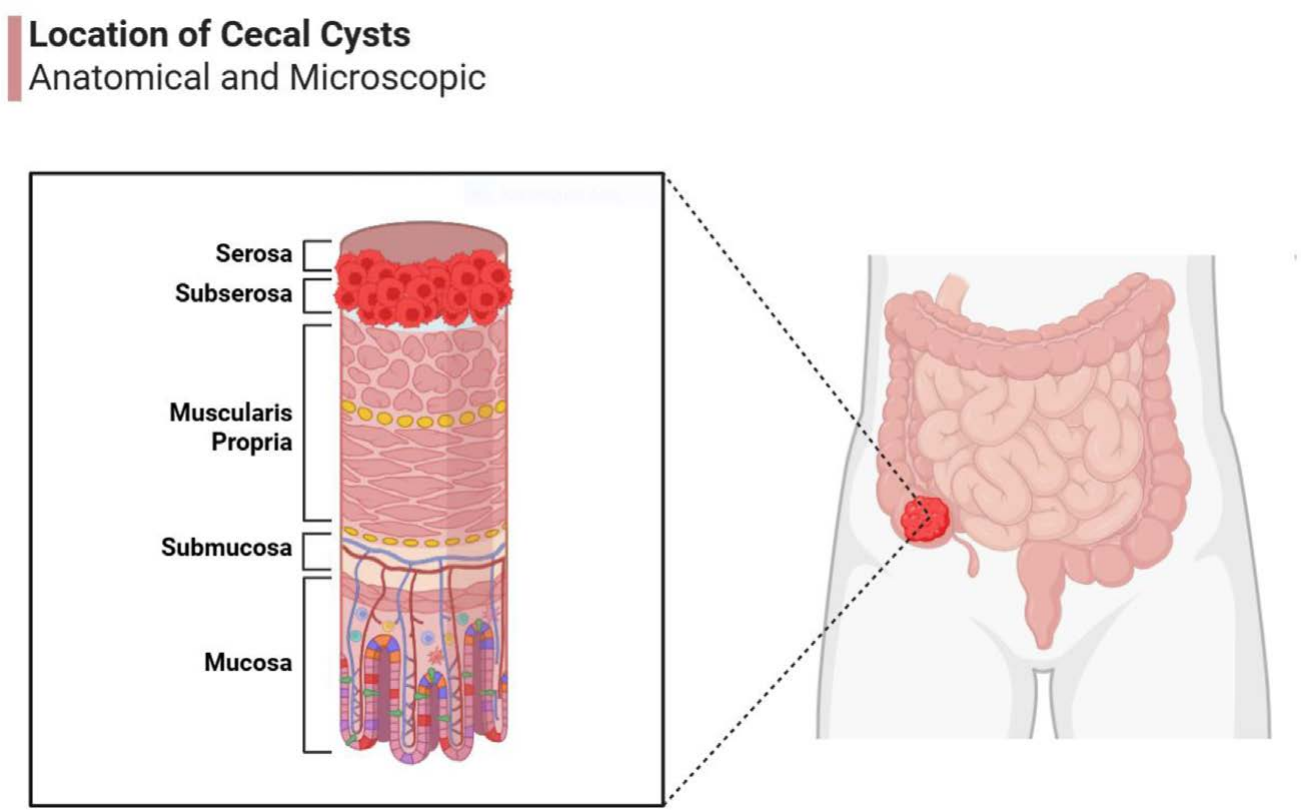
The anatomical and microscopic location of cecal dermoid cysts. Created in BioRender.

An alternative theory proposes that ectodermal implantation during the closure of the neural groove in embryogenesis may contribute to the formation of congenital dermoid cysts.^[5]^ However, this mechanism has been questioned by some researchers, particularly given the cecum’s location off the midline where the neural groove or other epithelial fusion lines are typically found. Another hypothesis suggests that dermoid cysts in the cecum may develop as the cecum reenters the abdominal cavity during intrauterine rotation. During this process, inclusion or fusion lines involving epidermal or dermal structures could contribute to cyst formation. The fact that most dermoid cysts in the cecum occur in a subserosal location supports this theory (Fig. 6).^[6,22]^

Regarding the demographic characteristics, dermoid cysts of the cecum can occur in both males and females, with a wide age range from pediatric to older patients. In terms of clinical presentation, these cysts are often asymptomatic, but they may present with chronic abdominal pain, gastrointestinal bleeding,^[20]^ or acutely with symptoms of intestinal obstruction.^[10]^ In some cases, a palpable abdominal mass may be detected during physical examination, and it may be tender^[13,17,21]^ or non-tender.^[7,12,14]^ On imaging, dermoid cysts typically present as well circumscribed cystic masses. However, their radiological features can resemble other conditions, such as lymphoma or duplication cysts, making diagnosis based solely on imaging challenging.^[6,16]^

Given the difficulty in making a definitive diagnosis without histopathological analysis, the preferred approach to management is surgical resection for both definitive diagnosis and appropriate treatment.^[17]^ This case highlights a rare and diagnostically challenging presentation of a benign dermoid cyst originating from the cecum, coexisting with acute appendicitis in a 14-year-old female. She presented with features suggestive of acute appendicitis, and preoperative imaging revealed a cystic mass along with lymphadenopathy, raising suspicion for a duplication cyst or neoplasm, thereby necessitating surgical exploration.

A cystic mass in the ileocecal region can have several differential diagnoses, including duplication cysts, mesenteric cysts, omental cysts, cystic lymphatic malformations, epidermoid cysts, and ovarian cysts.^[5]^ They may represent appendiceal mucocele, nonpancreatic pseudocysts, enteric duplication cysts, epidermoid cysts, or cystic teratomas. Additionally, because intramural cysts in the bowel are located near other structures such as the liver, the differential diagnosis may also involve hepatic cysts and choledochal cysts.^[7]^

While laparoscopic appendectomy is often sufficient for typical appendicitis, the presence of a suspicious mass led to the decision for oncological right hemicolectomy to ensure complete excision and histological assessment. This approach is consistent with previous reports recommending local excision or hemicolectomy, especially when malignancy cannot be ruled out, or when the lesion cannot be separated from the colon wall.^[5,9]^

Moreover, the use of laparoscopy in this case was both diagnostically and therapeutically effective, supporting its growing role in managing uncertain abdominal pathologies with minimal morbidity and faster recovery.^[1,7]^

Intraoperative management of a cecal mass in a pediatric patient requires individualized decision-making. Although a more limited resection, such as ileocecectomy, could be reasonable in selected cases, the presence of a discrete cecal lesion at the ileocecal junction with enlarged regional lymph nodes raised concern for an underlying malignant process. In our patient, this included lymphoma and neuroendocrine tumor, and to a lesser extent adenocarcinoma. Therefore, an oncologic right hemicolectomy was performed to ensure complete excision with appropriate lymphadenectomy. Frozen section could be considered in some centers; however, it was not feasible in our setting and may not reliably exclude all malignant entities, particularly lymphoma, which often requires permanent histology and immunohistochemistry for definitive diagnosis.

In contrast to the cases by Lao et al and Kirli et al,^[5,10]^ both of which described dermoid cysts without acute inflammation, our patient had a symptomatic presentation with leukocytosis, anemia, and concurrent acute appendicitis, with the latter symptom being the first case in literature. Lao et al described a congenital cecal dermoid cyst found incidentally in an asymptomatic 16-year-old, excised laparoscopically. Kirli et al reported the first pediatric case of an ileocecal dermoid cyst in an 11-year-old boy, who presented with abdominal pain. Both cases involved laparoscopic cystectomy, whereas our patient required a laparoscopic oncological right hemicolectomy due to appendicitis and the proximity of the cyst to the ileocecal junction. Histopathological examination in all 3 cases confirmed benign dermoid cysts, with similar features. Our case highlights the complexity of diagnosing and managing dermoid cysts when complicated by acute inflammatory conditions.

Finally, histopathological examination revealed a benign dermoid cyst and acute suppurative appendicitis with microscopic perforation, with no evidence of dysplasia or malignancy. This highlights the importance of considering a broad differential diagnosis in patients presenting with right lower abdominal pain, whether chronic or acute, particularly when unusual imaging findings are present. Early surgical intervention and histopathological evaluation remain critical for accurate diagnosis and management.

## 6. Conclusions

Cecal dermoid cysts are rare and when present alongside common conditions like appendicitis, they can confound the preoperative diagnosis and make it more challenging. In patients specifically pediatric patients, presenting with right lower abdominal pain and unclear radiological findings, it is important to keep in mind a wide range of differential diagnoses. This case highlights how helpful laparoscopy is, not just as a treatment method but for exploring what else might be going on. Since we couldn’t rule out serous differential diagnoses during surgery decision made to go ahead with a right hemicolectomy to be safe. At the end histopathology confirmed it was a benign dermoid cyst. This case demonstrates that unexpected intraoperative findings can occur, emphasizing that surgical adaptability is key to achieving the best patient outcomes.

## Author contributions

**Investigation:** Amagd Elsmani, Ali Toffaha.

**Methodology:** Hamza A. Abdul-Hafez.

**Writing – original draft:** Amagd Elsmani, Ali Toffaha, Hamza A. Abdul-Hafez, Wael Al-Ahmad, Mahmood Al-Dhaheri, Mahwish Khawar, Mohammed Yousif, Mohammed Kurer.

**Writing – review & editing:** Amagd Elsmani, Ali Toffaha, Hamza A. Abdul-Hafez, Wael Al-Ahmad, Mahmood Al-Dhaheri, Mahwish Khawar, Mohammed Yousif, Mohammed Kurer.
